# Enzyme Production Potential of *Penicillium oxalicum* M1816 and Its Application in Ferulic Acid Production

**DOI:** 10.3390/foods10112577

**Published:** 2021-10-26

**Authors:** Jing Zhang, Shuangping Liu, Hailong Sun, Zhengfei Jiang, Zhilei Zhou, Xiao Han, Yongxiang Zhou, Honggen Sun, Weibiao Zhou, Jian Mao

**Affiliations:** 1 National Engineering Laboratory for Cereal Fermentation Technology, School of Food Science and Technology, Jiangnan University, Wuxi 214122, China; 7160112032@stu.jiangnan.edu.cn (J.Z.); liushp@jiangnan.edu.cn (S.L.); 7160112067@stu.jiangnan.edu.cn (H.S.); 6200112032@stu.jiangnan.edu.cn (Z.J.); zlzhou@jiangnan.edu.cn (Z.Z.); hanxiao@jiangnan.edu.cn (X.H.); 2 Shaoxing Key Laboratory of Traditional Fermentation Food and Human Health, (Shaoxing) Industrial Technology Research Institute, Jiangnan University, Shaoxing 312000, China; weibiao@nus.edu.sg; 3 National Engineering Research Center of Huangjiu, Zhejiang Guyuelongshan Shaoxing Wine CO., LTD., Shaoxing 312000, China; Sb7799@163.com (Y.Z.); gylshg@163.com (H.S.); 4 Department of Food Science and Technology, National University of Singapore, Science Drive 2, Singapore 117542, Singapore

**Keywords:** ferulic acid, *Penicillium oxalicum*, wheat bran, *huangjiu* fermentation, whole genome

## Abstract

The present study focused on isolating an efficient enzyme production microorganism for ferulic acid (FA) production from wheat bran. A wild-type cellulase-, xylanase-, and feruloyl esterase-producing strain was isolated and identified as *Penicillium oxalicum* M1816. The genome was sequenced and assembled into 30.5 Mb containing 8301 predicted protein-coding genes. In total, 553 genes were associated with carbohydrate metabolism. Genomic CAZymes analysis indicated that *P. oxalicum* M1816, comprising 39 cellulolytic enzymes and 111 hemicellulases (including 5 feruloyl esterase genes), may play a vital role in wheat bran degradation and FA production. The crude enzyme of strain M1816 could release 1.85 ± 0.08 mg·g^−1^ FA from de-starched wheat bran (DSWB) at 12 h, which was significantly higher than other commercial enzymes. Meanwhile, when the strain M1816 was cultured in medium supplemented with DSWB, up to 92.89% of the total alkali-extractable FA was released. The process parameters of solid-state fermentation were optimized to enhance enzyme production. The optimized wheat bran *Qu* of *P. oxalicum* M1816 was applied to *huangjiu* fermentation, and the FA content was increased 12.4-fold compared to the control group. These results suggest that *P. oxalicum* M1816 is a good candidate for the development of fermented foods bio-fortified with FA.

## 1. Introduction

Wheat bran (WB) is a readily available byproduct produced by wheat processing. As an inexpensive by-product of agriculture, wheat bran consumption has excellent proven health benefits, such as improvement of gastrointestinal health, prevention of obesity and some types of cancer, and reduction of the risk of cardiovascular diseases and metabolic disorders [1,2,3]. However, due to the incomplete understanding of the nutritional value and function of wheat bran in the early days, it was mainly used as animal feed [4], which meant its value was not fully utilized, resulting in a serious waste of this resource. In recent years, WB has been increasingly used in human diets under the form of fermented and bakery production [5,6,7,8,9].

FA, a widely distributed hydroxycinnamic acid, is the major phenolic acid in the bran of wheat grain, accounting for up to 90% of the total phenolic acids [10]. FA is one of the most exploitable compounds in wheat bran [11]. Various scientific studies have proven that FA possesses great potential for health protection, such as antioxidant [12], anti-inflammatory [13], and antihypertensive [14] properties; and enhanced vascular function [15], among others. Surprisingly, FA may play a role in prophylaxis or improvement of COVID-19-associated symptoms, because of its antioxidant and anti-inflammatory properties [16]. Additionally, a growing number of studies have suggested that FA from wheat bran has higher biological activities than pure FA [17,18]. However, the low bioavailability of FA is one of the main factors that limits its widespread application, like in medical or functional food fields [19]. Fortunately, studies have shown that FA from wheat bran exhibits better bioavailability, compared to pure FA. An in vivo study in rats demonstrated that 43% of pure FA was excreted after 24 h, and only 2.6% of FA in wheat bran was excreted [20]. However, in wheat bran, up to 95% of FA is present in a bound form with the cell wall structures [20,21], which limits its bioavailability in the human body [15]. Considering these limitations, a priority strategy targets the release of bound FA in wheat bran before consumption, for an increased health effect due to the improved bioavailability of FA. To address this issue, many methods have been used to improve the release of FA from wheat bran, such as enzymatic treatments [11], and physical [22,23,24,25], chemical [26,27], and microbial fermentation [28] technology. Given the advantages of safety, costs, and environmental factors, microbial fermentation release of FA seems to be the most suitable method for food applications.

The release of FA from wheat bran is highly dependent on the hydrolysis degrees of the polysaccharide from the cell walls of wheat bran, as FA in the wheat bran is generally conjugated covalently with cellulose, hemicellulose, and lignin via ester bonds to form a dense network crosslinking structure [2]. Recent studies have shown that the coincubation of feruloyl esterase (FAE) with cellulase and xylanase could improve the saccharification of lignocellulosic biomass from wheat straw [29] and the degradation of polysaccharide can make FAE more efficient [30]. The synergism between cellulase and xylanase can break down the cell walls, which are mainly composed of lignin and cellulose, thus exposing the ester bond linked to FA in the cell wall of wheat bran. FAE is the key enzyme that specifically catalyzes the hydrolysis of ester linkages between FA and cell wall polysaccharides, and it is often used to measure the ability of a strain to produce FA [31]. Therefore, the combined action of FAE, cellulase, and xylanase can simultaneously cut off the ester bond between xylan and reduce the blocking effect between cellulose, thus increasing the release rate of FA. Wheat brans have been treated with feruloyl esterase, cellulase, and other enzymes by microbial fermentation to increase the content of FA, which has the advantages of mild reaction conditions, specificity to substrates, and environmental friendliness. Wheat *Qu* is made of wheat through solid-state fermentation. Wheat *Qu* is the primary liquefying and saccharifying agent for fermentation of *huangjiu*. *Huangjiu*, also known as Chinese rice wine, is a traditional fermented alcoholic beverage brewed directly from glutinous rice and wheat *Qu*. Previous studies indicated that wheat *Qu* harbors a variety of microorganisms, such as *Aspergillus*, *Penicillium*, *Pichia*, *Saccharopolyspora*, *Staphylococcus*, *Mucor*, *Rhizomucor*, *Lactobacillus*, *Bacillus*, and various enzymes, including amylases, glucoamylase, cellulase, proteases, and xylanase [32,33,34]. Therefore, wheat *Qu* could be a good source for screening a novel enzyme-producing microbe.

*Penicillium* species are important industrial filamentous fungi widely applied in food processing and fermentation, pharmaceuticals, biological degradation, and many other applicable fields. As reported, many *Penicillium* species have exhibited great potential for lignocellulose hydrolysis [35,36]. *P. oxalicum*, previously called *Penicillium decumbens*, has been used as cell factory for many industrially relevant enzymes’ production due to its outstanding capability of producing multienzymes, such as cellulases, glycoside hydrolase, and xylanases [35]. Therefore, *P. oxalicum* has excellent potential for the release of FA from wheat bran. The aim of the present study was to isolate, characterize, and identify the potent enzyme production strain, which can produce FA from wheat bran. In this study, a novel cellulase-, xylanase-, and feruloyl esterase-producing strain was isolated from *huangjiu* wheat *Qu* and identified as *P. oxalicum* M1816. Whole-genome sequencing will help to gain clear insights into the genomic basis of lignocellulosic biomass degradation and FA production. Further, the carbohydrate-active enzymes (CAZymes) were manually annotated and identified. In order to prove its capability to release FA from natural substrates, the efficiency of this strain and its crude enzyme to release FA from wheat bran was detected. To enhance the enzyme production by this strain, the process parameters of solid-state fermentation (SSF) were optimized. In addition, the potential for FA production of this strain in *huangjiu* fermentation was tested.

## 2. Materials and Methods

### 2.1. Screening and Identification of the Enzyme-Producing Strain

Wheat *Qu* were collected from a *huangjiu*-making factory (30°08′ N, 120°49′ E) in Shaoxing, Jiangsu Province, China in September, 2020. For microbial isolation on laboratory culture medium, wheat *Qu* samples were suspended in sterile distilled water, and serial dilutions were plated onto potato dextrose agar (PDA) medium (Solarbio, Beijing, China) for incubation at 28 °C for between 3 and 7 days. Single colonies were isolated, purified, and purified isolates preserved in 30% *v*/*v* glycerol at −80 °C. The isolated strains were spotted in triplicate on ethyl ferulate (EFA) agar [37], carboxymethylcellulose sodium (1% *w*/*v*, CMC-Na) agar, or xylan (0.5% *w*/*v*) agar selective solid media for the detection of feruloyl esterase (FAE), carboxymethyl cellulase (CMCase), and xylanase by qualitative and semi-quantitative agar spot methods [38]. The plates were incubated at 28 °C for 3 days to allow the secretion of enzymes. The enzymatic activity index (EI) was calculated as the ratio between the mean diameters of the substrates’ degradation halos and the average diameters of colonies [39]. All the chemicals used in the medium were reagent grade from Solarbio (Beijing, China) and Sinopharm (Shanghai, China).

### 2.2. SSF of Wheat Bran and Enzyme Assays

The 14 strains were cultivated in a solid-state fermentation medium (SFM) designed to enhance enzyme production, which consists of wheat bran and water at a ratio of 1:1, and were sterilized at 121 °C for 30 min. Spores were collected and adjusted to a concentration of 1 × 10^6^ spores/mL with sterile water. The SFM was inoculated with 5% (*v*/*v*) of spore suspension and incubated at 30 °C for 120 h under static conditions. Enzymes were extracted from SFM using 100 mL (1:10 ratio of bran solvent *w*/*v*) of sodium acetate buffer (NaAc, pH 4.6 100 mM) by shaking on a rotary shaker (180 rpm) at 30 °C for 1 h and filtered using Whatman filter paper (Whatman). The filtrate was centrifuged at 5000× *g* for 5 min at 4 °C using a high-speed refrigerated centrifuge (HIMAC CR21N, Hitachi, Tokyo, Japan). The crude enzymatic extracts (CEM1816) were stored at 4 °C for a maximum of 12 h until the enzymatic activity assays. FAE activity was estimated by measuring the release of FA in a reaction mixture containing 1 mL of enzyme solution and 1 mL of methyl ferulate (1 mM) in sodium acetate buffer (NaAc, pH 4.6 100 mM), at 30 °C and for 10 min. the reaction was terminated by boiling the reaction mixture for 5 min and the FA quantified by high performance liquid chromatography (HPLC). As controls, parallel experiments without enzyme and with heat-inactivated enzyme were carried out. CMCase activity was estimated by measuring the release of reducing sugars in a reaction mixture containing 0.5 mL of enzyme solution and 1.5 mL of carboxymethylcellulose sodium (CMC-Na, 1% *w*/*v*) in NaAc buffer (pH 4.6 100 mM), at 30 °C and for 10 min. The reaction was terminated by boiling the reaction mixture for 5 min. The reducing sugar was measured by the 3,5-dinitrosalicylic acid (DNS) method [40]. As controls, parallel experiments without enzyme and with heat-inactivated enzyme were carried out. Xylanase activity was estimated by measuring the release of reducing sugars in a reaction mixture containing 0.5 mL of enzyme solution and 1.5 mL of xylan (1% *w*/*v*) in NaAc buffer (pH 4.6 100 mM), at 30 °C and for 10 min. The reaction was terminated by boiling the reaction mixture for 5 min. The reducing sugar was measured by the DNS method. As controls, parallel experiments without enzyme and with heat-inactivated enzyme were carried out. All activities were expressed in international units (U) defined as 1 μmol of FA, glucose, or xylose produced per minute. Experiments were performed in triplicate and standard error was lower than 10% of the mean.

### 2.3. Morphology and Molecular Identification

The morphological characteristics of strain M1816 were studied using macroscopic and microscopic features. Macroscopic studies focused on the appearance, color, texture, and other physical features of the colony on PDA medium after incubation for 7 days. The hyphal morphology of strain M1816 was studied by bright-field microscopy (Nikon eclipse Ni, Tokyo, Japan) after staining with lacto-phenol cotton blue. The molecular characterization of strain M1816 was conducted using the sequence analysis of the ITS (internal transcribed spacer), LSU (large ribosomal subunit), and 18S (small subunit, SSU) regions with standard polymerase chain reaction (PCR) reaction. The used primers are listed in Table S1. ITS, LSU, and 18S sequences were aligned using ClustalW and phylogenetic trees were generated in MEGA-X software version 10.0.05, using the neighbor-joining method with 1000 bootstrap iterations.

### 2.4. Whole Genome Sequencing, Assembly, and Annotation

The genomic DNA of *P. oxalicum* M1816 was extracted using a genomic DNA extraction kit (Tiangen, Beijing, China). The integrity of the genomic DNA was confirmed by agarose gel electrophoresis and the DNA quantified by a Qubit^®^ 2.0 Fluorometer (Thermo Scientific). The whole genome of *P. oxalicum* M1816 was sequenced using the Nanopore PromethION platform (Oxford Nanopore Technologies Ltd., Oxford, UK) and Illumina Navaseq PE150 (Illumina, SanDiego, CA, USA) at the Novogene company (Tianjin, China). The sequencing reads were assembled de novo using Unicycler software (Version 0.4.8) [41]. The results of Illumina sequencing were used as a reference for single-base error correction of the Nanopore assembly results. Genes were predicted with Genewise software (version 2.4.1) using the default parameters [42]. Coding genes were annotated using basic local alignment search tool (BLAST) in the National Centre for Biotechnology Information (NCBI) NR database. Gene function was annotated using the euKaryotic Orthologous Group (KOG, http://www.ncbi.nlm.nih.gov/COG/, 13 May 2021) database, Kyoto Encyclopedia of Genes and Genomes (KEGG, http://www.genome.jp/kegg/, 10 June 2021) database, and Gene Ontology (GO, http://geneontology.org/, 14 June 2021) database.

### 2.5. CAZymes Identification

The identification and annotation of strain M1816 CAZymes were based on the Carbohydrate-active enzymes database (CAZy, http://www.cazy.org/, 2 July 2021) and performed on the automated Carbohydrate-active enzyme ANnotation (dbCAN2, http://cys.bios.niu.edu/dbCAN2, 21 July 2021) meta server [43], which integrates three state-of-the-art tools for CAZome (i.e., all CAZymes of a genome) annotation: (i) HMMER (biosequence analysis using profile hidden Markov models, http://www.hmmer.org/, 21 July 2021) search against the dbCAN HMM (hidden Markov model) database; (ii) DIAMOND search against the CAZy pre-annotated CAZyme sequence database; and (iii) Hotpep search against the conserved CAZyme short peptide database. This was followed by a further screening on the identified CAZyme based on the principle that the CAZy domains were recognized by at least two of the three tools (DIAMONDS, HMMER, and Hotpep) [44]. The genes predicted by dbCAN2 were manually verified by means of BLAST against the NR (non-redundant protein sequences) database. For each CAZyme, secretion was predicted by evaluating the presence of a secretion signal sequence using signal [45] (v. 4.1) with the default parameters.

### 2.6. Release of FA from DSWB

DSWB was prepared referring to a previously reported method with slight modification [46]. Briefly, 50 g of fresh wheat bran were soaked in 0.3% (*w*/*v*) potassium acetate at 95 °C with constant stirring for 30 min. Starch from wheat brans was washed away by distilled water and tested with iodine solution to monitor the completion of the reaction. Samples were oven dried at 105 °C for 3 h, ground, and filtered through a 60 mesh griddle for further analysis.

To assess the hydrolysis efficiency of the enzymes secreted by *P. oxalicum* M1816, enzymatic hydrolysis was carried out in reaction mixtures containing NaAc (pH 4.6 100 mM), 10 mL DSWB suspension (10 mg·mL^−1^), and enzyme (50 U/g DSWB, CEM1816, or commercial enzyme preparation) at 28 °C for 12 h. The reaction mixture was then terminated by boiling for 20 min and centrifuged at 10,000× *g* for 10 min. The supernatant was collected, filtered, and subsequently analyzed by HPLC (Waters, Milford, MA, USA) [29]. Wheat bran was subjected to enzymatic hydrolysis using commercial cellulase (Solarbio, Beijing, China), xylanase (Solarbio, Beijing, China), and feruloyl esterase (Megazyme, Bray, Ireland) as a positive control for comparison. Sample treated with the same procedure and with an equal amount of sterile water added was used as the blank group. The total alkali-extractable FA content of DSWB (10 mg) was measured following incubation of the substrate with 1 M NaOH (2 mL) for 4 h at 100 °C [46]. The hydrolysis product was centrifuged (10,000 rpm, 10 min) and supernatants were collected. The supernatant was filtered (0.22 μm) and measured using HPLC.

To detect the FA production by *P. oxalicum* M1816, 5 g DSWB were directly added into 100 mL of water before the autoclave process. *P. oxalicum* M1816 was inoculated in the medium cultured at 28 °C. The samples were taken out and analyzed at time intervals. The non-inoculated samples were used as the blank control. The percentage of FA released was calculated with the following formula: strain M1816 released FA content from per g DSWB/ total alkali-extractable FA content from per g DSWB.

### 2.7. Effects of SSF Conditions of Wheat Bran on Enzyme Production

The optimum fermentation condition is the key to successfully obtaining a high yield of fermentation products. The optimization fermentation conditions for maximum production of enzymes were investigated by SSF using wheat bran as the substrate, and the products were named wheat bran *Qu*. Four variables, namely fermentation time (h), moisture content (%), initial pH, and temperature (°C), the four key factors related to enzyme production of *P. oxalicum* M1816, were selected as independent variables to investigate the effect on enzyme production. The wheat bran solid medium containing wheat bran with water was autoclaved at 121 °C for 30 min. Medium was inoculated with spore suspension (2 × 10^5^ spores/g wheat bran). To determine the optimum fermentation time for enzyme production, strain M1816 was incubated for different time periods ranging from 0 to 168 h and incubated at 30 °C. To determine the optimum moisture content for enzyme production, the moisture content of the media was set from 40 to 100% (*v*/*w*) and incubated at 30 °C for 96 h. To determine the optimum initial pH for enzyme production, the initial pH of the media was adjusted to a value between 3.5 and 6.5 before sterilization using 1 M HCl and 1 M NaOH. The spores of strain M1816 were inoculated in the medium containing wheat bran with 80% water (*v*/*w*) and incubated at 30 °C for 96 h. From the above basis, to determine the optimum temperature for enzyme production, the fermentation temperature was optimized in the temperature range of 25 to 50 °C under pH 5.0. The crude enzyme extraction and CMCase, xylanase, and FAE activity determination methods were conducted by following the method described above. Additionally, the ability of the enzymes to release the bound FA from the complex natural DSWB was investigated. The activity assay was performed according to [46] with minor modifications. The reaction mixture contained 100 mg of DSWB and CEM1816 (1.0 mL) in a sodium acetate buffer (NaAc, pH 4.6 100 mM) at 30 °C and for 60 min. The reaction was stopped by putting the mixture in boiling water for 5 min. As controls, parallel experiments with heat-inactivated enzyme were carried out. After centrifugation (10,000× *g*, 15 min), the FA content of the supernatant was determined through HPLC. One unit (U) of DSWB activity was defined as the amount of enzymes required to liberate 1 µmol of FA from DSWB per minute under the assay conditions (NaAc, pH 4.6 100 Mm, 30 °C).

### 2.8. Application of P. oxalicum M1816 to Huangjiu Fermentation

*P. oxalicum* M1816 wheat bran *Qu* was prepared according to the optimized fermentation conditions. Lab-scale *huangjiu* fermentations were performed in a 5-L beaker with a 3-L working volume and produced with the techniques used by a *huangjiu* brewery in Shaoxing (Zhejiang Province, China) [47]. The addition of *P. oxalicum* M1816 wheat bran *Qu* in the experimental group was 2.0 g/100 g uncooked rice. The group without wheat bran *Qu* addition was the control group. The *huangjiu* fermentation was incubated at 30 °C for 100 h and then maintained at 16 °C for 300 h. *Huangjiu*’s reducing sugars were examined using the 3,5-dinitrosalicylic acid (DNS) method [40]. Titratable acidity, alcohol content, and amino acid nitrogen were measured using standard methods [48]. Amino acids, organic acids, and 4-vinylguaiacol (4-VG) were quantified as described before [49]. FA was quantified as described above. Further, 4-coumaric acid (4-CA), chlorogenic acid (CA), gallic acid (GA), vanillic acid (VA), caffeic acid (CAA), syringic acid (SA), and vanillic aldehyde (VAA) were quantified according to [31]. All experiments were performed in triplicate (*n* = 3).

### 2.9. Statistical Analyses

Data are expressed as mean ± standard error of the mean (SEM). All data were subjected to analysis of variance using GraphPad Prism software (Vision 8.02). Statistical significance was calculated using one-way analysis of variance, followed by Tukey tests. A *p* value of <0.05 was considered statistically significant.

## 3. Results and Discussion

### 3.1. Isolation, Screening, and Identification of the Enzyme-Producing Strain

**Figure 1:**
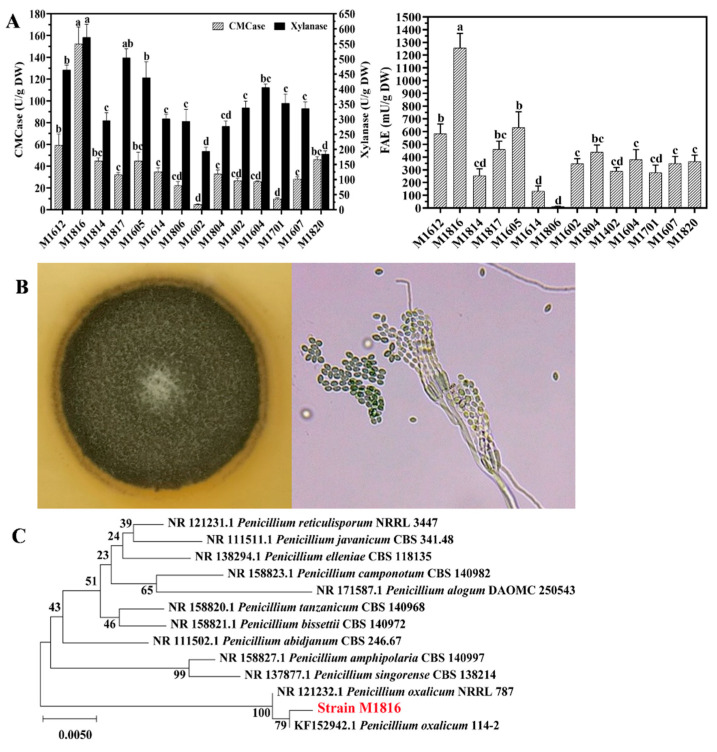
(**A**) The CMCase, xylanase, and feruloyl esterase (FAE) activities were measured from 14 strains inoculated for 120 h in wheat bran; DW, (dry weight). Different letters (a–d) indicate significant difference. (**B**) The colonial morphology of strain M1816 on potato dextrose agar (PDA) solid medium and morphological characteristics of hyphae and conidiophores by light microscope (400×). (**C**) Evolutionary relationships of strain M1816 were analyzed by the internal transcribed spacer (ITS) rRNA gene sequence with other homologous strains. The sequence accession numbers used for the phylogenetic analysis are shown before the species name. Alignments were calculated using ClustalW and Neighbor-Joining trees (Maximum Composite Likelihood method, 1000 bootstrap replicates) were constructed with MEGA-X.

A total of 14 strains that showed multiple (CMCase, xylanase, or FAE) enzymatic activities were isolated from wheat *Qu* samples (Table 1 and Figure 1). A microorganism is considered as a potential enzyme producer when it displays an enzymatic activity index ≥ 2.0 [50]. In this study, 28.57% of the selected microorganisms exhibited activity levels larger than 2.0 in the FAE screening plates, while 57.4% of them presented an EI ≥ 2.0 for xylanase (Table 1). Additionally, all the isolates showed lower CMCase EI to 2.0. The qualitative assay for enzymatic hydrolysis showed that strain M1816 and M1605 had a higher FAE and xylanase hydrolysis capacity. For further confirmation of the enzymatic activities of the isolates, CMCase, xylanase, and FAE analyses were performed, which revealed maximum enzyme activity for CMCase (152.19 ± 15.69 U/g DW), xylanase (571.75 ± 43.17 U/g DW), and FAE (1256.91 ± 65.29 mU/g DW) in strain M1816 (Figure 1A). The morphology of the strain M1816 was studied on PDA plates after 7 days of incubation at 28 °C. The colony was dark green, suborbicular, and had a dry surface. The hyphal morphology was observed under the Leica DM500 Light microscope (Leica, Wetzlar, Germany). Conidiophores were hyaline, smooth-walled, had three to four verticillate phialides on the top, and catevulate conidia in each phialide, forming a long chain. On the basis of the morphology, the strain was tentatively classified as belonging to the genus *Penicillium* (Figure 1B). The ITS, 18S, and LSU rRNA gene sequences of strain M1816 resulted in a closest hit of 100%, 99.70%, and 99.89% identity, respectively, to *P. oxalicum* 114-2 in the BLASTn searches on the NCBI NR database. The neighbor-joining tree showed that all obtained ITS rRNA sequences clustered together with the ITS rRNA gene sequences of *P. oxalicum* 114-2 (KF152942.1) and fell into the *Penicillium* clade (Figure 1C). The LSU and 18S sequences obtained in this study also fell into the same group with *P. oxalicum* 114-2 [35] (Figure S1). The strain M1816 was identified as *P. oxalicum* based on the morphological properties and ITS, LSU, and 18S rRNA sequence comparison. Recent studies have shown that the *Penicillium* genus can secrete a variety of highly active enzymes, such as cellulase, xylanase, β-glucosidase, lipase, inulinase, protease, and amylase, etc. [36,51]. These enzymes have good application prospects in food processing, industrial raw material preparation, and new energy manufacturing. The *P. oxalicum* M1816 is a newly isolated strain from fermented food, which was further used for optimized production of FA from wheat bran.

### 3.2. Genome Features and Gene Function Analysis of Strain M1816

The whole genome of strain M1816 was sequenced using two sequencing techniques, including the Nanopore PromethION system as the third-generation sequencing technology and Illumina Navaseq PE150 (paired-end) as the second-generation sequencing technology. Genome assembly of strain M1816 resulted in 9 contigs, with an N50 of 4.44 Mb. The M1816 genome was 30,536,533 bp in size and the GC content was 54.59% (Table S2). Genewise software was used to predict the whole genome sequence and 8301 coding sequences (CDS) were obtained. The total length of the CDS was 13,050,867 bp, accounting for 42.74% of the total genome length. Furthermore, 47 rRNAs (2 and 45 copies of 28S and 5S rRNAs, respectively) and 191 tRNAs were identified by rRNAmmer (Version 1.2) and tRNAscan-SE (Version 1.3.1), respectively [12,13].

**Figure 2:**
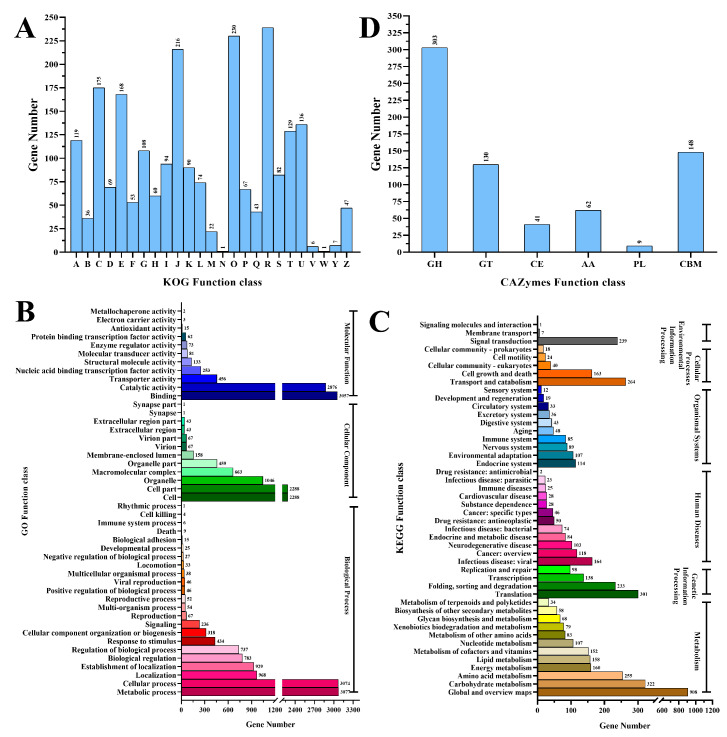
Gene function analysis of strain M1816’s (**A**) euKaryotic orthologous group (KOG) functional categories. (**B**) Gene ontology (GO) functional categories. (**C**) Kyoto encyclopedia of genes and genomes (KEGG) functional categories. (**D**) Carbohydrate-active enzymes database (CAZy) functional categories.

The functional annotation results showed that there are 2048, 5694, and 7629 genes annotated to KOG, GO, and KEGG, respectively (Figure 2). The coding proteins identified were classified into 25 functional categories, based on the classification of KOG (Figure 2A). Among the groups, general function prediction only (10.52%); post-translational modification, protein turnover, chaperones (10.12%); translation, ribosomal structure, and biogenesis (9.51%); energy production and conversion (7.70%); and amino acid transport and metabolism (7.39%) were the most abundant. The most abundant terms, according for 48.12% of all assigned GO terms (Figure 2B), include the metabolic process (12.25%), cellular process (12.24%), binding (12.17%), and catalytic activity (11.45%) subclasses, belonging to the biological process and molecular function classes, respectively. KEGG analysis showed that the global and overview maps (17.32%), carbohydrate metabolism (6.14%), translation (6.14%), and transport and catabolism (5.04%) were the most enriched pathways (Figure 2C).

A total of 553 CAZymes were annotated in *strain* M1816 (Figure 2D). Of the five functional CAZy classes, the most abundant class found in the *P. oxalicum* M1816 genome was glycoside hydrolases (GH), with a total number of 303 GH domains predicted in the genome. Apart from GHs, 130 domains of glycosyl transferases (GT), 22 domains of carbohydrate esterases (CE), 7 of polysaccharide lyases (PL), and 29 of auxiliary activity (AA) enzymes were found. A total of 62 carbohydrate-binding modules (CBMs), which are generally associated with GHs for better substrate binding, were also found in *P. oxalicum* M1816.

### 3.3. Potential of P. oxalicum M1816 to Release FA from Wheat Bran

To further investigate the potential of *P. oxalicum* M1816 to degrade wheat bran lignocellulose to produce FA, the putative encoding genes that may be involved in cellulolytic and hemicellulolytic deconstruction in CAZymes were identified (Table S3). Further analysis revealed that 150 CAZymes were predicted, specifically 39 cellulolytic enzymes and 111 hemicellulases. In wheat bran, FA is esterified to arabinose residues in the cell wall polysaccharides (CWPSs). Specifically, the CWPSs include 70% arabinoxylans, 24% cellulose, and 6% β-glucan [31]. Degradation of arabinoxylan is essential for the release of bound FA. Arabinoxylan requires the synergistic action of multiple enzymes for complete degradation. α-L-arabinofuranosidase (EC 3.2.1.55) and endo-1,5-α-L-arabinosidase (GH43, EC 3.2.1.99) remove arabinose substituents from the backbones of arabinoxylans (AX). Exo-α-L-1,5-arabinanase (GH93, EC 3.2.1-) can release arabinobiose from the nonreducing terminus of α-1,5-l-arabinan [52]. Xylan 1,4-β-xylosidase (GH3 and GH43, EC 3.2.1.37) cleave terminal xylose residues from the non-reducing ends of AX. Endo-1,4-β-xylanase (GH10, GH11, and GH30, EC 3.2.1.8) cleave AX into arabinoxylan-oligosaccharides and xylo-oligosaccharides. A total of 9 genes encoding α-L-arabinofuranosidase, 6 genes encoding xylan 1,4-β-xylosidase, 10 genes encoding endo-1,4-β-xylanase, 4 genes encoding endo-1,5-α-L-arabinosidase, and 3 genes encoding exo-α-L-1,5-arabinanase were detected in the genomes of *P. oxalicum* M1816.

Cellulose is the second main component of wheat bran, and its degree of degradation is important for the release of bound FA. Three groups of hydrolytic enzymes, cellulose 1,4-β-cellobiosidases (CBHs, EC 3.2.1.91, GH6, and GH9,), endoglucanases (EGs, EC 3.2.1.4, GH5, GH7, and GH45), and β-glucosidases (BGLs, EC 3.2.1.21, GH1, and GH3) were the classic enzymes involved in cellulose degradation [53]. Among the cellulolytic enzymes, 3 genes encoding CBHs, 8 genes encoding EGs, and 13 genes encoding BGLs were detected in the *P. oxalicum* M1816 genome. In addition, the genome of *P. oxalicum* M1816 also harbors another 15 genes, which encode enzymes that contribute to cellulose degradation. Lytic polysaccharide monooxygenase (LMPOs, formerly GH family 61) was also detected, which has been shown to catalyze the initial oxidative cleavage of recalcitrant cellulose. The LPMOs and cellulase synergy are beneficial for the degradation of large and highly resistant crystalline cellulose [54]. Moreover, the genes encoding xyloglucan hydrolase (XEG, EC 3.2.1.151, GH12), endo-β-1,6-glucanase (GH5 and GH30, EC 3.2.1.75), and β-*N*-acetylhexosaminidase (GH3, EC 3.2.1.52) have been observed to be involved in the hydrolysis of cellulose [55,56].

β-glucan is a non-starch polysaccharide found in the cell walls of wheat, consisting of repeated glucose residues linked by β-1,3 and β-1,4 glycosidic bonds. Hydrolysis of β-glucans is mainly carried out by four types of β-glucanases: β-1,3(4)-glucanase (GH16, GH81, and GH131, EC 3.2.1.6), endo-1,3-beta-D-glucosidase (GH17 and GH55, EC 3.2.1.39), endoglucanase (EC 3.2.1.4), and lichenase (CE1 and CE12, EC 3.2.1.73). Except EGs, the strain M1816 genome also possesses nine genes encoding β-1,3(4)-glucanase (A1069, A6094, A0698, A4246, A1189, and A7742), endo-1,3-beta-D-glucosidase (A0965 and A7572), and lichenase (A4549).

FAEs (EC 3.1.1.73), as the most critical enzyme that specifically breaks the ester bonds between arabinoxylan and FA, are often used as a characteristic enzyme in the degradation of FA. Based on the substrate specificity, whether to release diferulic acids, and the specificity of the protein sequence, FAEs can be divided into four types: A, B, C, and D [57]. A variety of FAEs have demonstrated beneficial effects in reducing recalcitrance and increasing biomass digestibility [58]. The *P. oxalicum* M1816 genome possesses five genes encoding FAE. Interestingly, the five FAEs harbor all four types. Gene A2202 (CE1) was 96.09% similar to the A-type FAE (accession number AGR85377.1) in *P. chrysogenum*. Gene A2266 (CE1) and A2649 (CE1) were 80.83% (accession number OOQ83522.1) and 75.89% (accession number OOQ83912.1) similar to the B-type FAE in *P. brasilianum*. Gene A0857 (CE12) and A4233 (CE1) encoded the type C (80.31% similarity, KAF3389293.1) and D (75.59% similarity, OKP02201.1), respectively.

While FAE can directly break the ester bonds between FA and polysaccharide, the key factor determining the amount of FA is the extent of wheat bran cell wall polysaccharide degradation. Additionally, the hydrolysis of the xylan backbone of wheat bran cell wall material (WBCW) enhances the accessibility of FAE to their substrates. Except for the genes mentioned above, the *P. oxalicum* M1816 genome also contains a number of genes encoding various enzymes including mannanase, galactosidase, esterase, pectinases, and others that cooperatively degrade WBCW. A total of 18 genes (α-galactosidase (GH27 and GH36, EC 3.2.1.22), β-mannosidase (GH2, EC 3.2.1.25), endo-1,4-β-mannosidase (GH5 and GH26, EC 3.2.1.78), and β-Galactosidase (GH2 and GH35, EC 3.2.1.23)) were predicted for galactomannan and mannan (second major component in hemicellulose) degradation. Hydrolysis of WBCW is also facilitated by esterases. Acetyl xylan esterase (CE1, CE2, and CE5, EC 3.1.1.72) and β-glucuronidase (GH79, EC 3.2.1.31) are the key enzymes attributed to the removal of the xylan side chains. In addition, various enzymes for hemicellulose hydrolysis, i.e., 1,3-β-glucosidase, endo-β-1,6-galactanase, α-glucuronidase, polygalacturonase, and others, were also present in abundance in the genome.

### 3.4. Release of FA from DSWB

**Figure 3:**
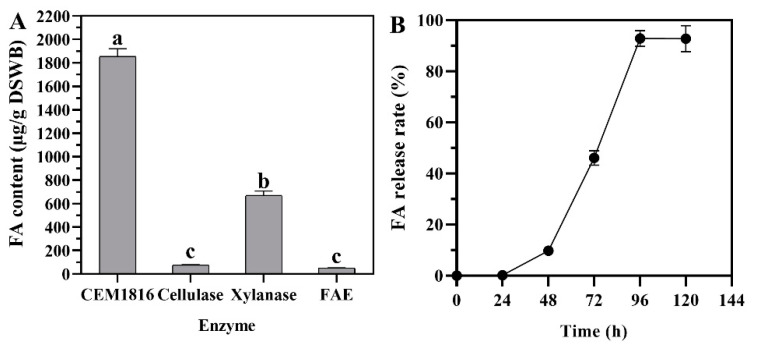
The releasing FA from de-starched wheat bran (DSWB). Different letters (a–c) indicate significant difference. (**A**) The ferulic acid (FA) production by CEM1816 and commercial enzyme preparation (cellulase, xylanase, FAE). (**B**) The time course of the produced FA by *P. oxalicum* M1816 cultured with DSWB.

Total alkali-extractable FA released from DSWB was determined to be 6.28 ± 0.47 mg·g^−1^. The yields of bound FA released from the complex natural substrates by CEM1816, cellulase, xylanase, and FAE were measured using DSWB as the substrate, respectively (Figure 3A). The results showed that CEM1816 released the highest amount of 29.46% of the total alkali-extractable FA (1.85 ± 0.08 mg·g^−1^) from DSWB, followed by xylanase (10.68%). Only 1.22% and 0.79% of total alkali-extractable FA was released by cellulase and FAE from DSWB, respectively. The result is similar to the results of previous studies [29,59], indicating that the enzymes secreted from strain M816 have excellent activity and potential application in FA production from complex natural substrates, like wheat bran. Several studies have reported on the release of FA from natural substrates by FAE from different microorganisms. Without the help of accessory enzymes, the purified FAE from the edible mushroom *Panus giganteus* could release 8.1% (17.6 μg/100 mg substrates) of the total FA from wheat bran [60]. The purified FarLcr from *Lactobacillus crispatus* could release a maximal amount of 199 µg FA from 0.2 g of DSWB [29]. This shows that we have screened a strain with good enzyme production performance. In addition, many studies have reported that only low levels, or no detectable levels, of free FA were released from wheat by FAE, cellulase, β-1,3-glucanase, and β-1,4-endoxylanase [13,47]. Many studies have shown that the synergy of multiple enzymes can significantly increase the release of FA [60,61]. In this study, the amount of FA released by CEM1816 was significantly higher than the other single enzymes. Additionally, CEM1816 was shown to have CMCase, xylanase, and FAE enzymatic activities in the above studies. This suggests that CEM1816 is a complex enzyme. As shown in Figure 3B, FA was gradually increased as time was prolonged. Finally, the release of FA reached a maximum after 96 h (92.89%). Therefore, strain M1816 as an enzyme-producing strain comes from fermented food that has great potential for degrading natural lignocellulose (like wheat bran) and producing FA.

### 3.5. Optimization of the SSF Conditions by P. oxalicum M1816 for Enzyme Production

**Figure 4:**
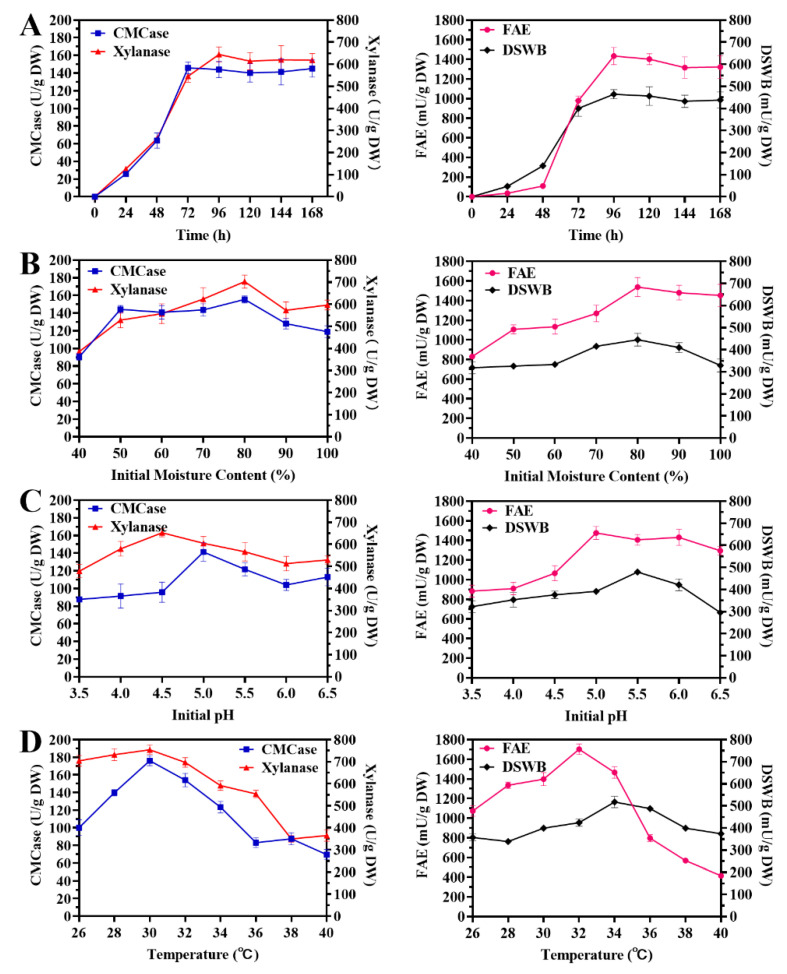
Effect of the fermentation conditions on CMCase, xylanase, FAE, and DSWB enzyme activities of *P. oxalicum* M1816. (**A**) Fermentation time (h). (**B**) Initial moisture content (%). (**C**) Initial pH. (**D**) Temperature (°C).

The fermentation time is a kinetic parameter with relevant importance for optimal enzyme production. The duration of fermentation will affect the growth rate of microorganisms and enzyme production. If the fermentation time is too short, microorganisms will grow rapidly and metabolism is vigorous, leading to lesser enzyme accumulation. If the fermentation time is too long, the nutrients in the fermentation medium will be exhausted and the secretase mechanism of microorganisms is inactivated, leading to low residual enzymatic activity [62]. The effects of the fermentation time on the activities of different enzymes are shown in Figure 4A. The CMCase, xylanase, and FAE activities increased significantly as the fermentation time increased. CMCase activity reached a maximum value of 145.87 ± 6.82 U/g DW at 72 h, while the xylanase and FAE activities reached a maximum value of 644.06 ± 34.14 and 1433.57 ± 87.14 mU/g DW at 96 h. After reaching the highest point, the activities of the three enzymes showed a fluctuating decreasing trend over time, which may be caused by the influence of the accumulated product of the fermentation on the enzyme. Using wheat bran as a substrate allows comprehensive evaluation of the enzyme-producing ability of the microorganism. After fermentation for 96 h, the enzymatic activity towards DSWB reached a maximum value of 464.32 ± 21.26 mU/g DW. In summary, the optimum fermentation time for enzyme production by *P. oxalicum* M1816 was determined to be 96 h.

Water is essential for microbial growth. The moisture content is one of the most critical factors that influences enzymatic production under SSF [63]. The effect of the initial moisture content on the activities of different enzymes is shown in Figure 4B. the CMCase enzymatic activity remained at a high level when the initial moisture content was between 50% and 90%, and the highest activity of 155.65 ± 4.15 U/g DW was observed with an 80% initial moisture content. The xylanase, FAE, and DSWB activities exhibited a trend of decreasing first and then rising as the moisture content increased and reached the highest point with an 80% initial moisture content. Therefore, the optimum initial moisture content for enzyme production by *P. oxalicum* M1816 was determined to be 80% (*v*/*v*).

The pH value is a major environmental factor controlling microbial growth [64]. The pH value can alter microbial cell membrane function to affect nutrient uptake and absorption, thus affecting the production of enzymes by microorganisms. The effects of the initial pH are shown in Figure 4C. The CMCase and xylanase activities increased firstly and then decreased with the change of initial pH, and reached the maximum activities at pH 5.0 and 4.5, respectively. The FAE and DSWB activities were relatively stable in the range of pH 5.0 to 6.0. As can be seen from the results of this experiment, an acidic environment is beneficial for the growth and production of enzymes by *P. oxalicum* M1816. This indicated *P. oxalicum* M1816 has an acid-tolerant nature. Therefore, the optimum initial pH for enzyme production by *P. oxalicum* M1816 was determined to be 5.0.

Temperature is generally considered the most important factor affecting metabolic functions and the growth and survival of microorganisms [65]. At a certain temperature, microorganisms become active and show accelerated growth and anabolism as temperatures increase. However, if the temperature is too high, it will affect the biomass and enzyme production of microorganisms. The effects of temperature are shown in Figure 4D. The CMCase and xylanase activities was relatively stable from 28 to 32 °C while at temperatures above 32 °C, the enzymatic activity reduced significantly. FAE and DSWB activities reached a maximum at 32 and 34 °C. The optimum fermentation temperature was thus determined to be 32 °C.

Overall, the optimum wheat bran *Qu* fermentation conditions of *P. oxalicum* M1816 were 96 h, initial moisture content of 80% (*v*/*w*), initial pH of 5.0, and 32 °C. After optimization, CMCase, xylanase, and FAE activities were enhanced by 1.17-, 1.32-, and 1.35-fold, respectively, compared to the non-optimized conditions. Antoine et al. studied the xylanase production condition by *Penicillium canescens* using soya oil cake under solid-state fermentation, and found that the optimal temperature and initial moisture of xylanase production were 30 °C and 80% [66]. This result is basically consistent with our results, and is consistent with the growth and metabolism characteristics of microorganisms of the *Penicillium* genus. Now, the optimization of growth conditions and the process has been attempted for solid fermentation to improve enzyme production. For example, Amadi et al. investigated the effects of the fermentation moisture ratio, pH, inoculum size, and incubation time on the cellulase and xylanase activities from Saccharomyces cerevisiae SCPW 17. After the optimization process, the cellulase and xylanase activities increased by 10.66% and 93.73%, respectively [67]. After the fermentation condition optimization, feruloyl esterase (BpFae) activities from *Burkholderia pyrrocinia* B1213 were increased 2.92 times higher than that obtained under optimal conditions using IPTG as the inducer [68]. Therefore, the study of the fermentation condition of enzyme production strains is of great significance to its application.

### 3.6. Production of FA by P. oxalicum M1816 and Its Application in Huangjiu Fermentation

**Figure 5:**
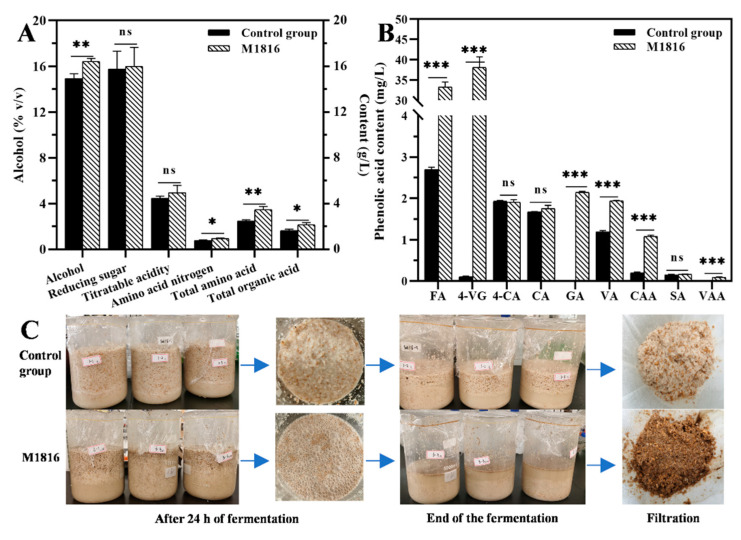
Physicochemical indexes (**A**) Alcohol, (**B**) phenolic acids content. (**C**) fermentation process of *huangjiu*. ns, not significant; *, *p* < 0.05 (significant); **, *p* < 0.01 (moderately significant); ***, *p* < 0.001 (highly significant).

To further determine the potential of *P. oxalicum* M1816 to degrade wheat bran and release FA in fermentation, the wheat bran *Qu* was scaled up to a 3-L *huangjiu* fermentation experiment. The physicochemical indexes and phenolic acid (FA and the other eight phenolic acids) content of fermented *huangjiu* are shown in Figure 5. All the physicochemical indexes of the fermented *huangjiu* complied with the Chinese national standard (GB/T 13662-2018). The alcohol, amino acid nitrogen, total amino acid, and total organic acid contents of the M1816 group were significantly higher than the control group (*p* < 0.05). There was no significant difference in the reducing sugar and titratable acidity contents (Figure 5A). *P. oxalicum* M1816 was able to increase the FA content in *huangjiu* by up to 33.30 mg/L, about 12.4-fold more than the control group (2.69 mg/L). In addition, compared with the control group, the contents of 4-VG, GA, VA, CAA, and VAA in the M1816 group *huangjiu* were also significantly improved (*p* < 0.05) (Figure 5B). Studies have shown that FA can be decarboxylated to 4-VG by thermal impact or through enzymatic decarboxylation during fermentation [69]. Further, 4-VG has a clove-like aroma, medicinal and phenolic flavor characteristics, and is an appreciable flavor constituent in certain beer styles, such as Belgian wheat and German Rauch beers [70]. FA was also a precursor for the conversion to VA and VAA, which are the main aroma components of natural vanilla flavor. As shown in Figure 5C, in the control group, after the fermentation, the remaining fermented mash floated on the surface, and relatively complete rice particles were visible. There is a lot of rice in the filtered fermented mash. In contrast, in the M1816 group, the remaining fermented mash sank to the bottom with no intact rice particles remaining, and very little rice remained in the filtered fermented mash. This indicated that *P. oxalicum* M1816 greatly promoted the degradation and utilization of rice, especially degradation of the cell wall. This result was consistent with the sequencing result of the *P. oxalicum* M1816 genome, which annotated a large number of genes encoding cellulose- and hemicellulose-degrading enzymes. Degradation of the rice cell wall leads to an increased fermentable sugar concentration and higher final alcohol content after fermentation. This is also the reason why the alcohol content of the M1816 group was higher than that of the control group. At the same time, phenolic acids produced by degradation of the rice cell wall also enter the *huangjiu*. Studies have shown that *Penicillium* can also produce a large number of proteases, which further promotes degradation of the protein in raw materials, thereby improving the content of amino acids [71].

## 4. Conclusions

*P. oxalicum* M1816 was isolated from *huangjiu* wheat *Qu*, a traditional Chinese fermented starter. Both genomic and experimental evidence demonstrates that *P. oxalicum* M1816 has the ability to produce FA from wheat bran. A comparison of wheat bran’s hydrolysis potential of the crude enzyme obtained from this strain with commercial preparations showed promising results and further increases the importance of *P. oxalicum* M1816 as a source of industrially relevant enzymes for wheat bran degradation. *P. oxalicum* M1816 can not only be a useful tool for FA production from wheat bran, but its fermentation byproducts (dietary fiber, phenolic acid, oligosaccharides, etc.) can also be applicable as a potential functional ingredient in the food industry for the improvement of health. *P. oxalicum* M1816 can be used for the development of functional food, and it can also be used in the animal feed industry to improve feed nutrition.

## Figures and Tables

**Table 1:** Enzymatic levels of the FAE, CMCase, and xylanase yielded by the isolated microorganisms.

Strains	FAE EI	CMCase EI	Xylanase EI
M1612	+	+	+++
M1816	++	+	+++
M1814	−	+	+++
M1817	−	+	++
M1605	++	+	++
M1614	−	+	++
M1806	+	+	++
M1602	−	+	++
M1804	+	+	+
M1402	+	+	+
M1604	++	+	+
M1701	+	+	+
M1607	+++	+	+
M1820	+	+	+

−, Enzymatic activity index (EI) is 0; +, EI between 1 and 2; ++, EI between 2 and 3; +++, EI between 3 and 4.

## Data Availability

Data is contained within the article.

## Supplementary Materials

The following are available online at https://www.mdpi.com/article/10.3390/foods10112577/s1, Figure S1: Evolutionary relationships of strain M1816 analyzed by LSU and 18S rRNA gene sequence with other homologous strains. The sequence accession numbers used for the phylogenetic analysis are shown before the species name. Alignments were calculated using ClustalW and Neighbor-Joining trees (Maximum Composite Likelihood method, 1000 bootstrap replicates) were constructed with MEGA-X, Table S1: Universal primer sequence, Table S2: Features of draft genome sequence of *P. oxalicum* M1816, Table S3: List of potential cellulolytic and hemicellulolytic genes in the genome of *P. oxalicum* M1816.
