# Supplementary material for: Enzyme Production Potential of Penicillium oxalicum M1816 and Its Application in Ferulic Acid Production
Source: Foods. 2021 Oct 26;10(11):2577. doi: 10.3390/foods10112577 (PMC8621443; doi:10.3390/foods10112577)
Supplement: Supplementary file 1 [file foods-10-02577-s001.zip › foods-1385365-supplementary.pdf]

Supplemental Figure

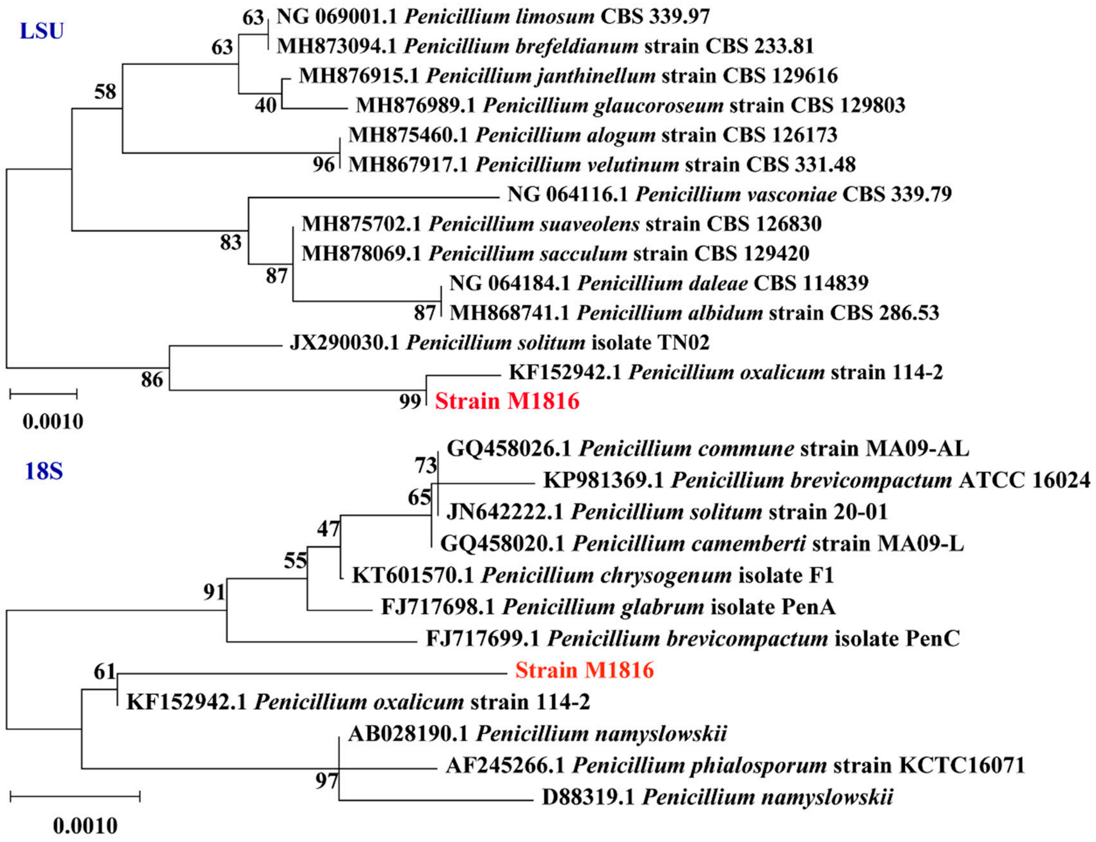

Figure S1. Evolutionary relationships of strain M1816 analyzed by LSU and 18S rRNA gene sequence with other homologous strains. The sequence accession numbers used for the phylogenetic analysis are shown before the species name. Alignments were calculated using ClustalW and Neighbor-Joining trees (Maximum Composite Likelihood method, 1000 bootstrap replicates) were constructed with MEGA-X.

Supplemental Table

Table S1. Universal primer sequence.

Amplified regions	Name	Squence（5’-3’）
ITS	ITS1	TCCGTAGGTGAACCTGCGG
	ITS4	TCCTCCGCTTATTGATATGC
LSU	LSUF	ACCCGCTGAACTTAAGC
	LSUR	TCCTGAGGGAAACTTCG
18S	NS1	GTAGTCATATGCTTGTCTC
	NS6	GCATCACAGACCTGTTATTGCCTC

Table S2. Features of draft genome sequence of *P. oxalicum* M1816.

Characteristics	*P. oxalicum* M1816
Genome size (bp)	30,536,533
Contigs	9
N50_Length(bp)	4,444,577
Protein-coding genes (CDS)	8,301
GC content (%)	54.59
Gene Length (bp)	13,050,867
Gene Average Length (bp)	1,572
Gene Internal Length (bp)	17,485,666
Number of tRNA genes	191
Number of rRNA genes	47

Table S3. List of potential cellulolytic and hemicellulolytic genes in the genome of *P. oxalicum* M1816.

Category	Predicted function	EC number	CAZyme families	Protein ID	Signal Peptide
Cellulose-degrading enzymes	Endoglucanase	3.2.1.4	GH5	A0042	N
			GH5+AA9+CBM1	A6487	Y
			GH5+CBM1	A2691	N
			GH5+CBM1	A5506	Y
			GH5+CBM1	A3679	Y
			GH5+CBM1	A0745	Y
			GH7+CBM1	A4283	Y
			GH45+CBM1	A4285	Y
	Cellulose 1,4-β-cellobiosidase	3.2.1.91	GH6+CBM1+CBM2	A5036	Y
			GH7+CBM1	A3458	Y
			GH7+CBM1	A4267	Y
	β-Glucosidase	3.2.1.21	GH1	A6549	N
			GH1	A0339	N
			GH1	A4001	N
			GH1	A7629	Y
			GH3	A0501	Y
			GH3+CBM1	A1149	N
			GH3+CBM1	A1311	N
			GH3+CBM1	A2507	Y
			GH3+CBM1	A5292	N

			GH3	A5963	N
			GH3+CBM1+CBM6	A7374	N
			GH3+CBM6	A7637	N
			GH3	A1633	N
	Lytic cellulose monooxygenase	1.14.99.54	AA9+CBM1	A3280	Y
			AA9+CBM1	A7645	N
			AA9+CBM1	A4123	N
			AA9+CBM1	A6654	N
	Lytic polysaccharide monooxygenases (LPMOs)		AA11	A0252	Y
			AA11	A4950	Y
			AA13+CBM20	A7561	N
	Xyloglucan hydrolase	3.2.1.151	GH12	A7702	Y
			GH12	A2502	N
			GH12+CBM1	A1166	Y
	Endo-β-1,6-glucanase	3.2.1.75	GH5	A6011	Y
			GH30	A7678	N
	β-N-Acetylhexosaminidase	3.2.1.52	GH3	A2707	N
			GH3	A7737	N
			GH3	A6927	N
Hemicellulose-degrading enzymes	Endo-1,4-β-xylanase	3.2.1.8	GH10+CBM1+CBM2+CBM22	A1628	Y

		GH10+CBM9	A6705	Y
		GH11	A3664	Y
		GH10+CBM1+CBM2+CBM22+CBM9	A2204	Y
		GH11	A3033	Y
		GH11	A0284	Y
		CE4+GH11+CBM1+CBM2+CBM36+CBM6	A7016	Y
		GH11+CBM1	A1364	Y
		GH30+CBM1	A6179	Y
		GH30	A5379	Y
Xylan 1,4-β-xylosidase	3.2.1.37	GH3	A2248	Y
		GH3	A6456	Y
		GH43	A1845	N
		GH43	A2137	N
		GH43	A4840	Y
		GH43	A6190	N
α-L-Arabinofuranosidase	3.2.1.55	GH43	A7824	N
		GH43	A4597	Y
		GH43+CBM2+CBM22+CBM42+CBM6	A8025	Y
		GH51	A5064	N
		GH51	A6276	N
		GH51	A7997	Y
		GH54+CBM13+CBM42	A0764	N
		GH62+CBM1+CBM13	A6201	Y

		GH62+CBM1+CBM13	A4313	Y
Endo-1,5-α-L-arabinosidase	3.2.1.99	GH43	A0770	Y
		GH43	A2462	N
		GH43	A4216	Y
		GH43	A7203	Y
Exo-α-L-1,5-arabinanase	3.2.1.-	GH93	A0101	Y
		GH93	A3022	N
		GH93	A2359	Y
Endo-1,3(4)-β-glucanase	3.2.1.6	GH16	A1069	Y
		GH16	A6094	N
		GH16	A0698	N
		GH16	A4246	Y
		GH81	A1189	N
		GH131+CBM1	A7742	N
Endo-1,3-beta-D-glucosidase	3.2.1.39	GH17	A0965	Y
		GH55	A7572	N
Lichenase	3.2.1.73	GH16	A4549	N
Feruloyl esterase	3.1.1.73	CE1	A2202	N
		CE1	A2266	N
		CE1	A2649	N
		CE1+CBM1+CBM2	A4233	Y
		CE12	A0857	Y
α-Galactosidase	3.2.1.22	GH27+AA9+CBM1+CBM13+CBM35+CBM51	A1534	N

		GH27	A5971	Y
		GH36	A3320	N
β-Mannosidase	3.2.1.25	GH2	A2299	N
		GH2	A3304	N
Endo-1,4-β-mannosidase	3.2.1.78	GH5	A0887	Y
		GH5+CBM1+CBM27	A2915	Y
		GH26+CBM35	A7703	N
α-1,2-Mannosidase		GH92	A2115	Y
		GH92	A4897	Y
		GH92	A4938	N
		GH92	A6562	Y
β-Galactosidase	3.2.1.23	GH2	A2155	N
		GH2+CBM42+CBM67	A1248	Y
		GH2+CBM51+CBM67	A5840	N
		GH35	A6282	Y
		GH35	A6701	Y
		GH35+GH43	A1514	Y
Acetylxylan esterase	3.1.1.72	CE1+CBM1	A0053	Y
		CE2	A7605	Y
		CE5+CBM1	A5891	Y
β-Glucuronidase	3.2.1.31	GH79	A5025	N
1,3-β-Glucosidase	3.2.1.58	GH5	A2234	N
		GH5	A1249	Y
		GH5	A4150	Y
		GH55	A6309	Y
Endo-β-1,6-galactanase	3.2.1.164	GH30	A8032	Y

Endo-β-1,4-galactanase	3.2.1.89	GH53	A1461	Y
		GH53	A8027	N
α-Glucuronidase	3.2.1.139	GH67	A5378	N
SUN family β-glucosidase	3.2.1.-	GH132	A2684	N
		GH132	A6251	N
Polygalacturonase	3.2.1.15	GH28	A0867	Y
		GH28	A4274	Y
		GH28	A5010	Y
		GH28	A5874	Y
		GH28	A5224	Y
		GH28	A5275	Y
		GH28	A7063	N
Rhamnogalacturonan hydrolase	3.2.1.171	GH28	A0061	N
		GH28	A3937	Y
		GH28	A1356	N
D-4,5-unsaturated β-glucuronyl hydrolase	3.2.1.-	GH88	A1152	N
Unsaturated rhamnogalacturonyl hydrolase	3.2.1.172	GH105	A7620	N
Rhamnogalacturonan acetylesterase	3.1.1.86	CE12	A7083	Y
		CE12	A0799	N
Pectin methylesterase	3.1.1.11	CE8	A7401	Y
		CE8	A2300	N

		CE8+GH28	A3052	Y
		CE8+GH28	A7272	Y
Tannase	3.1.1.20	Not assigned	A2268	N
		Not assigned	A7471	N
Pectin lyase	4.2.2.10	PL1	A4913	Y
		PL1	A7456	Y
Pectate lyase	4.2.2.2	PL1	A8024	Y
Rhamnogalacturonan endolyase	4.2.2.23	PL4	A7531	Y
		PL4	A6367	Y
		PL4	A4532	N
L-threo-3-deoxy-hexylosonate aldolase	4.1.2.54	Not assigned	A3717	N
L-galactonate dehydratase	4.2.1.146	Not assigned	A5529	N
α-L-rhamnosidase	3.2.1.40	GH78	A1357	N
		GH78	A1649	N
		GH78+CBM67	A5413	N
		GH78+CBM67	A7691	N
α-L-fucosidase	3.2.1.51	GH95+CBM13+CBM32+CBM51	A4002	N
